# Sex differences in methylation profiles are apparent in medulloblastoma, particularly among SHH tumors

**DOI:** 10.3389/fonc.2023.1113121

**Published:** 2023-03-24

**Authors:** Rachel M. Moss, Natali Sorajja, Lauren J. Mills, Christopher L. Moertel, Thanh T. Hoang, Logan G. Spector, David A. Largaespada, Lindsay A. Williams

**Affiliations:** ^1^ Division of Epidemiology & Clinical Research, Department of Pediatrics, University of Minnesota, Minneapolis, MN, United States; ^2^ Bioinformatics and Computational Biology, University of Minnesota, Minneapolis, MN, United States; ^3^ Macalester College, St. Paul, MN, United States; ^4^ Masonic Cancer Center, University of Minnesota, Minneapolis, MN, United States; ^5^ Pediatric Hematology and Oncology, Department of Pediatrics, University of Minnesota, Minneapolis, MN, United States; ^6^ Brain Tumor Program, University of Minnesota, Minneapolis, MN, United States; ^7^ Department of Pediatrics, Division of Hematology-Oncology, Baylor College of Medicine, Houston, TX, United States; ^8^ Dan L. Duncan Comprehensive Cancer Center, Baylor College of Medicine, Houston, TX, United States; ^9^ Cancer and Hematology Center, Texas Children’s Hospital, Houston, TX, United States; ^10^ Department of Pediatrics, University of Minnesota, Minneapolis, MN, United States; ^11^ Department of Genetics, Cell Biology and Development, University of Minnesota School of Medicine, Minneapolis, MN, United States; ^12^ Center for Genome Engineering, University of Minnesota, Minneapolis, MN, United States

**Keywords:** medulloblastoma, sex differences, methylation, survival, pediatric and young adult cancer

## Abstract

**Background:**

Medulloblastoma, the most common malignant pediatric brain tumor, displays marked sex differences in prevalence of the four main molecular subgroups: SHH, WNT, Group 3 and Group 4. Males are more frequently diagnosed with SHH, Group 3 and 4 tumors, which have worse prognoses than WNT tumors. Little is known about sex differences in methylation profiles within subgroups.

**Methods:**

Using publicly available methylation data (Illumina HumanMethylation450K array), we compared beta values for males versus females. Differentially methylated positions (DMP) by sex within medulloblastoma subgroups were identified on the autosomes. DMPs were mapped to genes and Reactome pathway analysis was run by subgroup. Kaplan-Meier survival curves (Log-Rank p-values) were assessed for each sex within subgroup. *MethylCIBERSORT* was used to investigate the tumor microenvironment using deconvolution to estimate the abundances of immune cell types using DNA methylation data.

**Results:**

There were statistically significant differences in sex by medulloblastoma subgroups (chi-squared p-value=0.00004): Group 3 (n=144; 65% male), Group 4 (n=326; 67% male), SHH (n=223; 57% male) and WNT (n=70; 41% male). Females had worse survival than males for SHH (p-value=0.02). DMPs by sex were identified within subgroups: SHH (n=131), Group 4 (n=29), Group 3 (n=19), and WNT (n=16) and validated in an independent dataset. Unsupervised hierarchical clustering showed that sex-DMPs in SHH did not correlate with other tumor attributes. Ten genes with sex DMPs (*RFTN1*, *C1orf103*, *FKBP1B*, *COL25A1*, *NPDC1*, *B3GNT1*, *FOXN3*, *RNASEH2C*, *TLE1*, and *PHF17)* were shared across subgroups. Significant pathways (p<0.05) associated with DMPs were identified for SHH (n=22) and Group 4 (n=4) and included signaling pathways for RET proto-oncogene, advanced glycosylation end product receptor, regulation of KIT, neurotrophic receptors, NOTCH, and TGF-β. In SHH, we identified DMPs in four genes (*CDK6*, *COL25A1*, *MMP16*, *PRIM2)* that encode proteins which are the target of therapies in clinical trials for other cancers. There were few sex differences in immune cell composition within tumor subgroups.

**Conclusion:**

There are sexually dimorphic methylation profiles for SHH medulloblastoma where survival differences were observed. Sex-specific therapies in medulloblastoma may impact outcomes.

## 1 Introduction

Medulloblastoma is the most common malignant pediatric brain tumor (1) affecting 400 United States (US) children each year (2). Medulloblastoma is comprised of four main molecular subgroups (3) that are prognostic with sonic hedgehog (SHH), Group 3 and Group 4 tumors associated with worse prognoses than wingless (WNT) tumors (4). We have shown there to be a male predominance in medulloblastoma incidence and risk in the US and around the globe in population-based studies (5–7). Unfortunately, with the recent use of these subgroups for prognosis they are almost entirely lacking in population-based and registry studies. As such, we must rely on clinical studies to understand sex differences in outcomes. From clinical studies, there are documented differences in the distribution of medulloblastoma subgroups by sex, with males more frequently diagnosed with the high-risk Groups 3 and 4 subgroups (8). While there are differences in survival between subgroups, little is known about sex differences in survival within subgroups. Further, there is no work examining sex differences in the genomic landscape of medulloblastoma, which may have significant implications for treatment and outcomes.

Sex differences in brain tumor development and progression are multifactorial and depend on the sex chromosome complement (9), immune regulation (10), and intrinsic differences in methylation that begin at conception through the life course (11). Based on the male excess in brain tumor diagnoses at all ages, it is unlikely that sex hormones are a main driving force mechanistically (12). Sex differences in epigenetics as measured by methylation have been documented throughout various organ systems in the body including the brain (13). DNA hypomethylation is often associated with carcinogenesis. Therefore, sex differences in methylation patterns could impact medulloblastoma formation and growth (13). Further, as methylation plays an integral role in brain tumor development and progression, it is important to identify sex differences in methylation profiles, which may help us understand etiology of the disease and identify potential therapeutic targets. Therefore, using publicly available medulloblastoma methylation data (14, 15), we aimed to identify sex differences in methylation profiles within subgroups.

## 2 Materials and methods

### 2.1 Data source

Using publicly available DNA methylation data collected by Cavalli et al. (2017) (14), 763 primary medulloblastoma samples were considered in our main analysis. Briefly, patient samples were collected from a number of treatment institutions including The Hospital for Sick Children, Children’s Hospital of Philadelphia, the Mayo Clinic and others (14). Patient samples were only included in the initial study if their medulloblastoma diagnosis required surgical resection. Flash frozen tissues were obtained, DNA from these samples was extracted, and methylation values were determined using Illumina Infinium HumanMethylation450 BeadChips.

### 2.2 Survival analyses

Chi-squared tests were performed to identify sex differences in the distribution of medulloblastoma subgroups. Fisher’s Exact tests (p<0.05) were performed to test for subgroup-specific sex differences in the distribution of selected covariates including age at diagnosis (years; 0-<5, 5-<10, 10-<15, 15-<20, ≥20), histology (classic, desmoplastic, large-cell anaplastic, medulloblastoma with extensive nodularity), metastasis (yes, no), and vital status (dead, alive). Kaplan-Meier survival curves were constructed and Log-Rank p-values were utilized to compare 12.5-year overall survival differences between sexes. Figures were created using *survminor* (R v4.0.2). Cox proportional hazards models were used to estimate hazard ratios (HR) and 95% confidence intervals (95% CI) as the measure of association between sex and death within each subgroup adjusting for covariates listed above (SAS v9.4). No violation of the proportional hazard’s assumption was detected as determined by entering a sex and time interaction term in the model.

### 2.3 Quality control and sex prediction

The R *minfi* package (Bioconductor v.3.12) (16) was used to convert the original methylation array experiment to an RGChannel Set object and perform quality control. The RGChannel Set contains the raw intensities of each probe. To detect and remove probes with unreliable signals, a function producing a detection p-value for each probe in every sample was run. Probes that returned p-values above 0.01 were removed from the analysis (n=44,069). The RGChannel Set was processed before differential methylation analysis by undergoing functional normalization, thus transforming it into a Genomic Ratio Set. Functional normalization was used as it is commonly applied to datasets with different tissues and cell types. Probes containing single nucleotide polymorphisms (SNPs) at either the CpG interrogation and/or at the single nucleotide extension were removed (n=60,291). Similarly, we removed non-specific, cross-reactive probes previously found to hybridize to autosomal and sex chromosomes by Chen et al. (2013) (n=106,931) (17).

Sex of each patient’s tissue was predicted by observing the median intensity of the X and Y chromosome probes (getSex() *minfi*). Any samples with discordant sex from the clinical information and predicted sex from the methylation data were dropped from the analysis (n=55). The sex chromosomes were excluded from all differentially methylated position analyses.

### 2.4 Differential methylation and mapping of differentially methylated positions to genes

After removal of the aforementioned probes, beta values, a standard estimate of the percentage methylation as a ratio of methylation probe fluorescent signal intensity to total probe signal (*Beta = Meth/(Meth + Unmeth + offset)* of the remaining probes were retrieved using the *minfi* function getBeta on the functionally normalized Genomic Ratio Set. The *bumphunter* package was used to run a multivariate model to examine differentially methylated regions, or “bumps”, by sex and subgroup, including adjustment for age at diagnosis (years; 0-<5, 5-<10, 10-<15, 15-<20, ≥20). A cut-off value of 0.05 was used in the model and 500 permutations were run. Differentially methylated positions (DMPs) between sex (male-female) were identified using the lmFit function in custom R code, with adjustment for age at diagnosis as in the DMR analysis. Differential methylation analysis by sex was done within each medulloblastoma subgroup (SHH, Group 3, Group 4 and WNT) and subsequently within each SHH subtype (SHH_alpha, SHH_beta, SHH_gamma, SHH_delta) as defined in Cavalli et al. (2017) (14). Significant DMP’s (Benjamini Hochberg adjusted p-value <0.05) within each subgroup were mapped to their nearest genes using the gene annotations from the 450k probe annotation information for genome hg19 (**Supplemental Tables 1**–**5**). Samples missing age data were excluded from this analysis (n=34). Additional healthy adult cerebellum samples detailed in the CNV analysis below were processed in the same manner to identify sex-DMPs in non-diseased tissues. Heatmaps were created using *ComplexHeatmap* and the Venn diagram of overlapping genes in subgroups was created using Venny (18).

### 2.5 Reactome pathway analysis

Reactome pathway analysis was used to identify biologic pathways over-represented by genes that had significant DMPs by sex for each medulloblastoma subgroup (p-value <0.05; date accessed: 10/15/2022). Pathways composed of ≥2 genes that had a significant p-value were included herein. IPA BioProfiler was used to identify chemotherapies available in clinical trials for other human cancers for the genes that contained DMPs by sex (date accessed: 2/24/2021).

### 2.6 Immune cell profiling based on methylation values

The *MethylCIBERSORT* R package (version 0.2.0) (19) and CIBERSORTx (https://cibersortx.stanford.edu/) (20) were used to investigate the tumor microenvironment using deconvolution to estimate the abundances of immune cell types using DNA methylation data. The *Stromal_v2* reference from MethylCIBERSORT was used as the methylation signature matrix file. Input matrices of beta values for the reference probes in the signature matrix were created as percentages and uploaded to CIBERSORTx with the signature matrix. CIBERSORTx was run in absolute mode using 1,000 permutations without quantile normalization.

### 2.7 Copy number variation analysis

CNV analysis was performed using the *conumee* R package on raw methylation data (IDAT files) from the Illumina 450k methylation arrays to confirm that differentially methylated regions or positions were not copy number driven. [http://bioconductor.org/packages/conumee/] *Conumee* requires control data for analysis. Therefore, we downloaded a publicly available dataset that measured DNA methylation using Illumina’s 450K array in non-demented control brain tissue of the cerebellum (n=179, https://www.ncbi.nlm.nih.gov/geo/query/acc.cgi?acc=GSE134379). *Conumee* normalizes the combined intensity values of the methylated and unmethylated probes of each CpG site using these controls (representing genomes with no copy number alterations). Surrounding probes are then combined to create bins of a minimum size and probe number (default values and *conumee exclude_regions* data were used) prior to segmentation into clusters of the same state of variation in the number of copies *via* the circular binary segmentation algorithm. Segment tables were created for all samples and segment means for male versus female samples within subgroups at each DMP/gene/chromosome region of interest were calculated using pairwise Wilcoxon Rank Sum test.

### 2.8 Validation analysis

DMP analysis was validated in an independent cohort using publicly available DNA methylation data collected by Schwalbe et al. (2017) (15). This set consisted of 428 clinically annotated primary medulloblastoma samples. These tumor samples were part of a UK Children’s Cancer and Leukaemia Group (CCLG) study with approval from Newcastle North Tyneside Research Ethics committee (heretofore referred to as the Newcastle cohort). The Newcastle patient samples were also tested using Illumina HumanMethylation 450 BeadChips. Quality control and sex prediction were performed as described above. Samples were removed that had lower median intensities and did not cluster using *minfi plotQC* (badSampleCutoff=10.5, n=21). Additionally, 36 samples were removed as they were discordant between reported and predicted sex. As subgroup classification data was not available for the Newcastle cohort, the R package *MethPed* was used to perform classification (R package version 1.24.0.) (21). Ten additional samples were removed based on classification to categories outside of medulloblastoma by this algorithm, leaving a total of 361 samples used in the DMP validation analysis (**Table 1A**). Survival data, metastasis, and vital status were unavailable for the Newcastle cohort.

**Table 1A:** Cavalli case demographics and clinical characteristics stratified by sex and medulloblastoma subgroup.

	SHH		WNT		Group 3		Group 4		Chi-square p-value
	Females	Males	Females	Males	Females	Males	Females	Males	
	N (%)	N (%)	N (%)	N (%)	N (%)	N (%)	N (%)	N (%)	
**Sex**	86 (40.4)	127 (59.6)	38 (56.7)	29 (43.3)	36 (28.1)	92 (71.9)	89 (29.7)	211 (70.3)	**0.00004**
Age at diagnosis									
**0-<5**	33 (40.2)	42 (34.1)	2 (5.7)	0 (0)	10 (28.6)	43 (50.6)	15 (17.2)	33 (16.4)	
**5-<10**	15 (18.3)	23 (18.7)	10 (28.6)	10 (38.5)	19 (54.3)	30 (35.3)	47 (54.0)	86 (42.8)	
**10-<15**	5 (6.1)	11 (8.9)	13 (37.1)	4 (15.4)	6 (17.1)	7 (8.2)	21 (24.1)	60 (29.9)	
**15-<20**	8 (9.8)	11 (8.9)	8 (22.9)	4 (15.4)	0 (0.0)	1 (1.2)	2 (2.3)	18 (9.0)	
**≥20**	21 (25.6)	36 (29.3)	2 (5.7)	8 (30.8)	0 (0.0)	4 (4.7)	2 (2.3)	4 (2.0)	
**missing**	4	4	3	3	1	7	2	10	
**Fisher’s p-value**		0.88		**0.03**		0.05		0.15	
Histology									
**Classic**	32 (44.4)	43 (42.2)	24 (88.9)	14 (70.0)	18 (64.3)	42 (67.7)	59 (81.9)	126 (78.3)	
**Desmoplastic**	27 (37.5)	42 (41.2)	1 (3.7)	4 (20.0)	3 (10.7)	3 (4.8)	8 (11.1)	14 (8.7)	
**LCA**	7 (9.7)	13 (12.7)	2 (7.4)	2 (10.0)	7 (25.0)	15 (24.2)	5 (6.9)	16 (9.9)	
**MBEN**	6 (8.3)	4 (3.9)	0 (0.0)	0 (0.0)	0 (0.0)	2 (3.2)	0 (0.0)	5 (3.1)	
**missing**	14	25	11	9	8	30	17	50	
**Fisher’s p-value**		0.60		0.19		0.65		0.44	
Metastasis									
**No**	52 (82.5)	79 (85.9)	24 (92.3)	17 (85.0)	17 (60.7)	43 (60.6)	48 (63.2)	92 (58.2)	
**Yes**	11 (17.5)	13 (14.1)	2 (7.7)	3 (15.0)	11 (39.3)	28 (39.4)	28 (36.8)	66 (41.8)	
**missing**	23	23	23	23	23	23	23	23	
**Fisher’s p-value**		0.65		0.64		1.00		0.48	
Vital Status									
**Alive**	48 (70.6)	87 (84.5)	33 (97.1)	24 (92.3)	19 (67.9)	44 (58.7)	59 (72.8)	121 (70.8)	
**Deceased**	20 (29.4)	16 (15.5)	1 (2.9)	2 (7.7)	9 (32.1)	31 (41.3)	22 (27.2)	50 (29.2)	
**missing**	18	24	4	3	8	17	8	40	
**Fisher’s p-value**		**0.036**		0.57		0.50		0.77	

Statistically significant p-values (<0.05) are in bold.

## 3 Results

**Figure 1:**
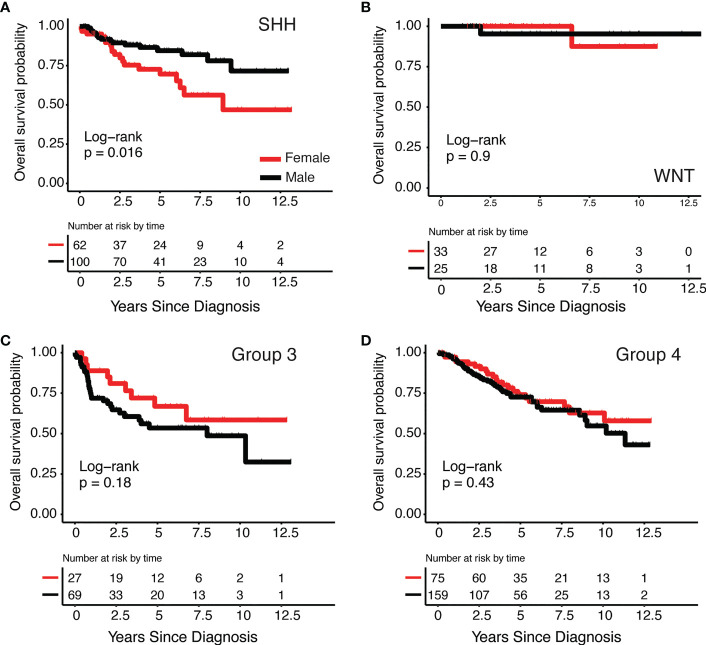
Kaplan-Meier survival curves by medulloblastoma subgroup and sex **(A)** SHH, **(B)** WNT, **(C)** Group 3 and **(D)** Group 4.

There were 708 cases included in this analysis: SHH (N=213, 59.6% male), WNT (N= 67, 43.3% male), Group 3 (N= 128, 71.9% male), and Group 4 (N=300, 70.3% male) (**Table 1B**). We observed significant 12.5-year overall survival differences by sex in the SHH subgroup (**Figure 1A**) non-significant differences were observed in other subgroups, (**Figures 1B–D**) such that females had lower survival than males (Log-Rank p=0.016). Using Cox proportional hazards models adjusted for age at diagnosis, histology, and metastasis, SHH females had nearly three times the risk of death compared to males (hazard ratio: 2.89, 95% CI: 1.29-6.24). There were no significant differences in survival between males and females in the other three subgroups.

**Table 1B:** Newcastle case demographics and clinical characteristics stratified by sex and medulloblastoma subgroup.

	SHH		WNT		Group 3		Group 4		Chi-square p-value
	Females	Males	Females	Males	Females	Males	Females	Males	
	N (%)	N (%)	N (%)	N (%)	N (%)	N (%)	N (%)	N (%)	
**Sex**	35 (39.8)	53 (60.2)	16 (55.2)	13 (44.8)	31 (32.3)	65 (67.7)	47 (31.8)	101 (68.2)	0.075
Age at Diagnosis									
**0-<5**	23 (65.7)	31 (58.5)	0 (0)	2 (15.4)	18 (58.1)	37 (56.9)	11 (23.4)	16 (15.8)	
**5-<10**	7 (20.0)	10 (18.9)	11 (68.)	4 (30.8)	10 (32.3)	25 (38.5)	24 (51.1)	61 (60.4)	
**10-<15**	4 (11.4)	10 (18.9)	5 (31.3)	6 (46.2)	2 (6.5)	2 (3.1)	12 (25.5)	20 (19.8)	
**15-<20**	1 (2.9)	2 (3.8)	0 (0)	1 (7.7)	1 (3.2)	1 (1.5)	0 (0)	4 (4.0)	
**>=20**	0 (0)	0 (0)	0 (0)	0 (0)	0 (0)	0 (0)	0 (0)	0 (0)	
**Fisher’s p-value**		0.85		0.08		0.69		0.33	
Histology									
**Classic**	7 (20.0)	17 (32.1)	11 (68.8)	10 (76.9)	22 (71.0)	41 (63.1)	35 (74.5)	81 (80.2)	
**Desmoplastic**	12 (34.3)	19 (35.8)	0 (0)	1 (7.7)	0 (0)	2 (3.1)	4 (8.5)	5 (5.0)	
**LCA**	8 (22.9)	8 (15.1)	3 (18.8)	0 (0)	4 (12.9)	18 (27.7)	3 (6.4)	8 (7.9)	
**MBEN**	6 (17.1)	6 (11.3)	0 (0)	0 (0)	0 (0)	0 (0)	0 (0)	0 (0)	
**NOS**	2 (5.)	3 (5.7)	2 (12.5)	2 (15.4)	5 (16.1)	4 (6.2)	5 (10.6)	7 (6.9)	
**Fisher’s p-value**		0.65		0.33		0.16		0.65	

**Figure 2:**
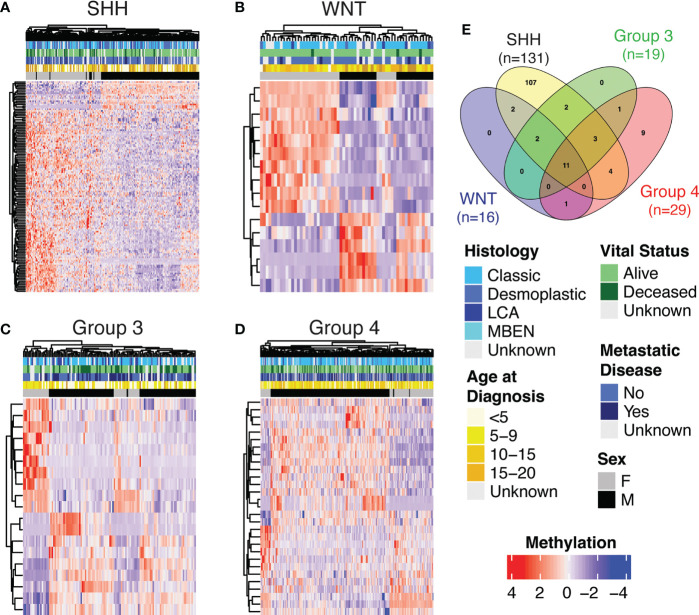
Heatmap showing methylation levels (row-scaled β-values) of statistically significantly differentially methylated positions (DMPs) by sex (adjusted p<0.05) from the autosomes in Cavalli cohort within subgroup **(A)** SHH, **(B)** WNT, **(C)** Group 3 and **(D)** Group 4. **(E)** The number of genes that contained a DMP by sex for each subgroup and the overlap of those gene sets.

We used the *bumphunter* package to run a multivariate model to examine sex differences globally by sex and subgroup and found 12 statistically significant DMPs (**Supplemental Table 1**). After finding this small number of DMPs globally, we then investigated subgroup-specific differentially methylated positions by analysis within each subgroup of tumor as medulloblastoma subgroups are molecularly and prognostically distinct (4). We observed statistically significant sex differences in DNA methylation within each subgroup (**Supplemental Tables 2**–**5**). SHH had the highest number of DMPs by sex (n=131), followed by Group 4 (n=29), Group 3 (n=19), and WNT (n=16). Ten genes had statistically significant DMPs by sex in all subgroups: *RFTN1*, *C1orf103*, *FKBP1B*, *COL25A1*, *NPDC1*, *B3GNT1*, *FOXN3*, *RNASEH2C*, *TLE1*, and *PHF17*. We performed unsupervised hierarchical clustering using significant DMPs by sex within each subgroup (**Figures 2A**–**D**). Clustering of samples using these DMPs was independent of other clinically relevant characteristics such as histology and vital status. The SHH group showed the strongest clustering by sex-DMPs (**Figure 2A**).

CNV analysis was performed to confirm that differentially methylated regions or positions were not copy number driven (**Supplemental Tables 2**–**5**). Less than ten percent of sex-DMPs in each subgroup were statistically significantly different when we intersected CNV segments with DMP locations and compared mean values for males versus females (SHH=9.9%, Group 4 = 3.5%, Group3 = 0%, WNT=0%). The same approach confirms that in the SHH subgroup samples there is no statistically different level of amplification at *GLI2*, *MYCN*, and *TP53* or 14q and 17p chromosome arm loss between sexes (results not shown).

To validate our sex-DMPs, we performed subgroup analysis on the validation set arising from the Newcastle cases, which resulted in a smaller, but consistent group of statistically significant sex-DMPs in each subgroup. Again, SHH had the highest number of DMPs by sex (n=11), followed by Group 4 (n=5), Group 3 (n=4), and WNT (n=2) (**Supplemental Tables 6**–**9**). All but one of these subgroup sex-DMPs, the gene *LTK* in the WNT subgroup, were already found in the corresponding Cavalli subgroup analysis. One gene, *RFTN1*, was a statistically significant DMP by sex in all subgroups. Unsupervised hierarchical clustering of subgroup samples using significant sex-DMPs demonstrates that the clustering is independent of other clinically relevant characteristics, specifically age at diagnosis and histology, and the SHH group shows the strongest clustering by sex (**Supplementary Figure 1**).

Statistically significant pathways resulting from the Cavalli sex-DMPs for each subgroup are presented in **Table 2A**. To explore tumor specific pathways, we removed genes associated with sex-DMPs that overlap with those found in the control cerebellum brain samples (**Supplementary Table 10**) and repeated the pathway analysis (**Table 2B**). No pathways were identified in WNT or Group 3 in either analysis. The top two pathways in Group 4 (both overall and tumor only sex-DMPs) were activation of *HOX* genes during differentiation and anterior *HOX* genes in hindbrain development during early embryogenesis. Both are also found in the top pathways of SHH using all sex-DMPs. Tumor specific sex-DMP pathways in SHH included G alpha (s) signaling events, diseases associated with N-glycosylation of proteins, telomere C-strand (lagging strand) synthesis, and interleukin-1 signaling. The top four pathways in this SHH tumor-specific pathway analysis overlaped with the overall sex-DMP pathway analysis. The top pathway in the non-tumor-specific sex-DMPs of SHH is YAP1- and WWTR1 (TAZ)-stimulated gene expression. Other top pathways identified in SHH include signaling and loss of function of TGF-β receptor in cancer, SOS-mediated signaling, signal attenuation, signaling in RET, and advanced glycosylation end product receptor. Using the IPA BioProfiler, we identified four genes that encode proteins that are the target of therapies approved or in clinical trials for other human cancers that contained sex-DMPs including *CDK6*, *COL25A1*, *MMP16*, *PRIM2* in SHH and *COL25A1* in WNT and Group 4.

**Table 2A:** Reactome pathway analysis for enriched pathways (p<0.05) comprised of at least two genes with significant differences in methylation by sex for each medulloblastoma subgroup*.

Reactome Pathway Name	pValue	Submitted entities found
SHH		
YAP1- and WWTR1 (TAZ)-stimulated gene expression	0.00005	TEAD4;RUNX2
Loss of Function of TGFBR1 in Cancer	0.00283	SMAD2;FKBP1B
Signaling by TGF-beta Receptor Complex in Cancer	0.00462	SMAD2;FKBP1B
SOS-mediated signalling	0.00566	GRB2;IRS2
Signal attenuation	0.00936	GRB2;IRS2
RET signaling	0.01247	GRB2;IRS2;SHANK3
Advanced glycosylation endproduct receptor signaling	0.01387	PRKCSH;CAPZA1
Regulation of RUNX2 expression and activity	0.01409	RUNX2;RBX1
Regulation of KIT signaling	0.01731	GRB2;SOCS6
Pre-NOTCH Transcription and Translation	0.01772	NOTCH3;H3F3A
Erythropoietin activates RAS	0.01915	GRB2;IRS2
WNT5A-dependent internalization of FZD4	0.02107	ARRB2;AP2B1
Signaling by NTRK1 (TRKA)	0.02211	ADCYAP1;NAB1;GRB2;IRS2;AP2B1
Signaling by NOTCH	0.02602	NOTCH3;TLE1;H3F3A;ARRB2;RBX1
Intrinsic Pathway for Apoptosis	0.03480	YWHAQ;APAF1
Signaling by NTRKs	0.03823	ADCYAP1;NAB1;GRB2;IRS2;AP2B1
Pre-NOTCH Expression and Processing	0.03872	NOTCH3;H3F3A
mTORC1-mediated signalling	0.03900	FKBP1B;EIF4EBP1
RAB geranylgeranylation	0.04045	RAB23;RAB12;RAB33B
Activation of anterior HOX genes in hindbrain development during early embryogenesis	0.04083	H3F3A;HOXC4
Activation of HOX genes during differentiation	0.04083	H3F3A;HOXC4
SARS-CoV-1-host interactions	0.04300	YWHAQ;NAB1;FKBP1B
Group 4		
Activation of HOX genes during differentiation	0.00020	HOXC4;POLR2L
Activation of anterior HOX genes in hindbrain development during early embryogenesis	0.00020	HOXC4;POLR2L
Organelle biogenesis and maintenance	0.00149	ATP5J;CSNK1D;RAB11FIP3;GABPA
Mitochondrial biogenesis	0.00405	ATP5J;GABPA

*No pathways were detected for WNT or Group 3.

**Table 2B:** Reactome pathway analysis for enriched pathways (p<0.05) comprised of at least two genes with significant differences in methylation by sex for each medulloblastoma subgroup (without overlap with non-tumor cerebellum sex-DMPs) *.

Reactome Pathway Name	pValue	Submitted entities found
SHH		
Regulation of RUNX2 expression and activity	0.00223	RUNX2;RBX1
Advanced glycosylation endproduct receptor signaling	0.00503	PRKCSH;CAPZA1
Transcriptional regulation by RUNX2	0.01599	RUNX2;RBX1
Signaling by NOTCH	0.02767	NOTCH3;ARRB2;RBX1
G alpha (s) signalling events	0.03564	ADCYAP1;PDE2A;ARRB2;TAPBP
Diseases associated with N-glycosylation of proteins	0.03669	ALG9;ALG6
Telomere C-strand (Lagging Strand) Synthesis	0.04111	PRIM2;WRN
Interleukin-1 signaling	0.04889	IKBIP;RBX1
Group 4		
Activation of anterior HOX genes in hindbrain development during early embryogenesis	0.00001	HOXC4;POLR2L
Activation of HOX genes during differentiation	0.00001	HOXC4;POLR2L
Mitochondrial biogenesis	0.00031	ATP5J;GABPA
Organelle biogenesis and maintenance	0.00036	ATP5J;RAB11FIP3;GABPA
Respiratory electron transport, ATP synthesis by chemiosmotic coupling, and heat production by uncoupling proteins.	0.01109	ATP5J;COX5B
The citric acid (TCA) cycle and respiratory electron transport	0.02549	ATP5J;COX5B
VxPx cargo-targeting to cilium	0.02602	RAB11FIP3
Developmental Biology	0.04028	HOXC4;POLR2L

*No pathways were detected for WNT or Group 3.

As the SHH subgroup had significant overall survival differences by sex, we explored sex-DMPs in the four clinically and cytogenetically distinct SHH subtype groups: SHH alpha (N = 59, 61.0% male), SHH beta (N = 32, 46.9% male), SHH gamma (N = 45, 55.6% male), and SHH delta (N = 69, 68.1% male). Although smaller sample sizes in these subtypes limited our ability to find methylation differences due to sex, we observed that SHH delta subtype had the largest number of sex-DMPs within SHH delta subtypes (n=38). SHH delta likely drove many of the differences by sex found in SHH overall (**Supplementary Tables 11**–**14**; **Supplementary Figure 2**).

**Figure 3:**
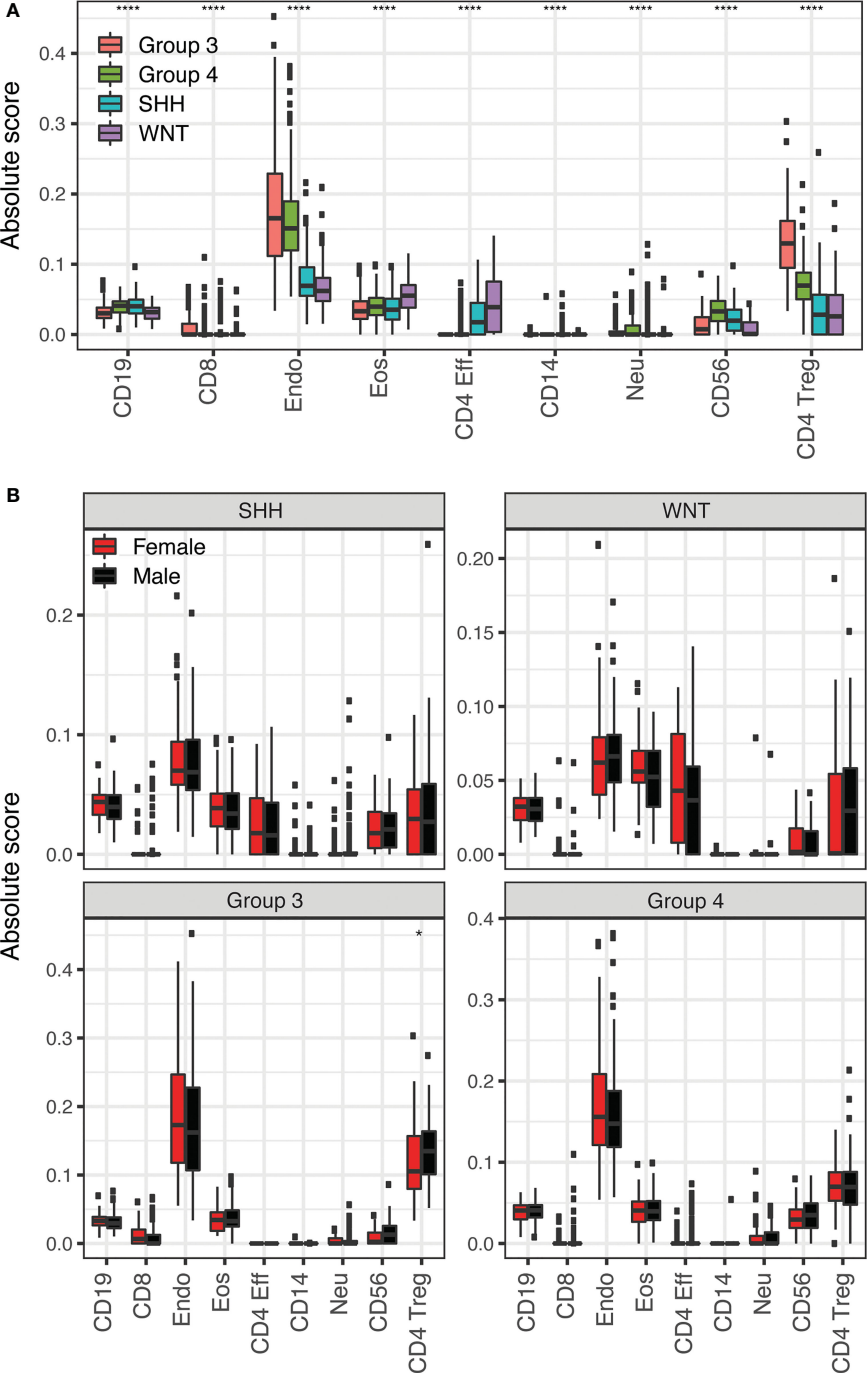
**(A)** Application of the MethylCIBERSORT stromal signature matrix to deconvolution of 708 medulloblastoma 450K methylation arrays using CIBERSORT. Boxplots compare medulloblastoma subgroup means for each cell type. Significance is calculated using pairwise Wilcoxon Rank Sum test with p-values (*p <= 0.05; ****: p <= 0.0001). The symbol is shown for the highest significance between two different subgroups for each cell type. **(B)** Boxplots comparing female (red) and male (black) means for each cell type in each medulloblastoma subgroup as labeled.

Due to the complex nature of the tumor microenvironment and role of the immune system in tumor development, medulloblastoma subgroup and sex differences in tumor immune cell composition were assessed using MethylCIBERSORT. Comparisons of medulloblastoma subgroups overall for each immune cell type assessed in the MethylCIBERSORT deconvolution pipeline show statistically significant differences in distribution between at least two subgroups in each cell type (all p<0.0001, **Figure 3A**) though the absolute scores of these cell types were low overall. Within subgroup comparisons of males versus females cell type composition showed statistically significant differences in regulatory T cells in Group 3 (**Figure 3B**). No other subgroups displayed statistically significant sex differences in immune cell types identified in MethylCIBERSORT (**Figure 3B**).

## 4 Discussion

From 708 primary medulloblastoma samples, we identified statistically significant sex differences in survival in the SHH subgroup, with females demonstrating worse long-term survival than males. There were no survival differences between sexes in the Group 3, Group 4, or WNT subgroups. We identified sex differences in methylation within the four subgroups and SHH had the highest number of sex-DMPs (n=131), followed by Group 4 (n=29), Group 3 (n=19), and WNT (n=16). In our validation cohort, sex-DMPs were identified in smaller numbers, but were largely the same as those seen in the Cavalli data further strengthening the evidence of sex differences in methylation in medulloblastoma. Unsupervised hierarchical clustering based on sex-DMPs did not appear to be driven by any other clinically-relevant factors. The strongest sex driven clustering was observed in the SHH subgroup, which comprise approximately 30% of medulloblastomas in general (1, 4). These findings suggest there are true sex differences in DMPs within each subgroup that are independent of important clinical factors.

After mapping the sex-DMPs to the nearest gene, there were ten genes shared between all four subgroups. Nine of these ten genes were also found as sex-DMPs in the healthy adult cerebellum tissue analysis, suggesting more global sex differences in brain methylation that may be not disease- or age- specific. The genes identified in all four subgroups were *RFTN1*, *C1orf103*, *FKBP1B*, *COL25A1*, *NPDC1*, *B3GNT1*, *FOXN3*, *RNASEH2C*, *TLE1*, and *PHF17.* Of these, *RFTN1*, *COL25A1*, *TLE1*, and *RNASEH2C* have been found to have sex differences in methylation in leukocytes (22), which are known to impact tumor maintenance (23) and be regulated by sex hormones (24, 25). Remaining genes from this list are involved in various neuron-related processes, such as *FKBP1B* and neuronal aging (26), *NPDC1* and neuronal differentiation (27), and *FOXN3* and neuronal activation (28). Sex differences in brain development have been reported extensively in the psychiatric literature such that males not only have larger brain volumes by approximately 10% (12, 29) they also have a larger volume of gray matter (30). Gray matter is composed of various neuronal cell types (30) and may contribute to the sex differences in brain tumor incidence we see in populations as well as the sex differences we observed herein. We did not find that sex-DMPs corresponded to numerous sex differences in CNVs again suggesting a true role for sex-DMPs in medulloblastoma. Collectively, these findings suggest that sexually dimorphic epigenetic regulation of these genes may underlie medulloblastoma etiology more broadly and may operate through leukocyte-mediated mechanisms and neuronal development.

In our analysis, we also identified biologic pathways from the lists of sex-DMPs that may be sexually dimorphic in medulloblastomas. The top pathway in SHH is YAP1- and WWTR1 (TAZ)-stimulated gene expression. YAP1 and WWTR1 are both transcriptional co-activators regulated *via* HIPPO signaling with transcriptional targets crucial to cell proliferation and apoptosis. HIPPO signaling has previously been associated with pediatric cancers, including a known interaction with Sonic Hedgehog that upregulates the nuclear localization of *YAP* (31). Several top pathways identified using only tumor specific sex-DMPs in SHH were also found in the top SHH pathways using all sex-DMPs, including regulation of *RUNX2* expression and activity. *RUNX2* has been previously implicated in SHH tumorigenesis (32). Additional pathways from the tumor-specific sex-DMPs in SHH include G α (s) signaling events and diseases associated with N-glycosylation of proteins. The G α (s) signaling pathway can suppress SHH tumorigenesis through negative regulation of the Hippo pathway. The top two pathways in Group 4 are activation of *HOX* genes during differentiation and anterior *HOX* genes in hindbrain development during early embryogenesis, and both are also found in the top pathways of SHH. *HOX* genes are critical to embryonic development, with their expression accompanied by specific epigenetic states with noted changes to DNA methylation in other brain tumors (33). Other top pathways identified in SHH include signaling and loss of function of TGF-β receptor in cancer, SOS-mediated signaling, signal attenuation, and signaling in RET and advanced glycosylation end product receptor. TGF-β secretion has been documented in medulloblastomas and TGF-β pathway activity is potentially a predictor of survival in SHH-driven medulloblastomas (34). We then went on to identify four genes from our IPA BioProfiler analysis that had current targeted therapies approved for use in other cancers: *CDK6*, *COL25A1*, *MMP16*, *PRIM2*, which highlights the potential sex-specific utility of these genes and their encoded proteins as therapeutic targets for future study.

Population-based studies show incidence rates for medulloblastoma vary by sex both within the United States and around the world, with males more frequently diagnosed and male-to-female incidence rate ratios ranging between 1.4-2.2 (5, 6, 24); however, these studies often lack modern subgroup classifications. Based on findings from clinical-based studies, male-to-female ratios differ between molecular subgroups, with WNT and SHH groups showing approximately equal male and female distributions, but Group 3 and Group 4 medulloblastoma comprised with about twice as many males (11, 35). In our study, we observed an excess of males in SHH medulloblastoma rather than a 1:1 distribution by sex. Characterizing the sex ratio in population-based studies with genomic samples is critical.

Ten-year survival rates by sex in medulloblastoma are similar among all ages in studies, including our previous publication (24, 36), while five-year survival rates in children aged 0-19 indicate a lower risk of death in females than males (HR: 0.79) (37). Group 3 and 4 subgroup tumors are often found to have poorer survival than WNT tumors across the age-spectrum (35) yet we did not observe survival differences by sex in our study within these groups. Conversely, we observed that females in our study had worse survival than males for SHH tumors, which have an approximate 5-year survival of 50-75% depending on *TP53* mutation status (1, 38). Unfortunately, we could not evaluate *TP53* mutation status in our SHH tumors as we were unable to match samples to their mutation status based on the publicly available data (14), but there was no indication of sex differences in *TP53* CNV in our analyses. However, we did observe that SHH females had a 5-year survival of approximately 60% in our study suggesting the association between *TP53* mutation status and sex should be evaluated in future studies. Age-stratified analyses among SHH tumors where *TP53* mutation is thought to occur more commonly in children (8) were non-informative in our analysis likely due to sample size limitations (results not shown). The male excess in brain tumor incidence is not confined to medulloblastoma. There is a male excess in gliomas (39) and significantly decreased overall survival in males for recurrent gliomas (40). Notably, Johansen et al. (2020) found genome-wide differences in DNA methylation by sex, with distinct patterns in glioma molecular subgroups as we have observed in medulloblastoma (41).

Sex differences in brain tumor genomics and epigenetics have been discussed extensively by Rubin and colleagues (11, 12). Sex can influence tumorigenesis through the sex chromosome complement, direct hormone action, and epigenetic disparities (11). Given that the molecular subgroups of medulloblastoma are suspected to arise from different cells of origin (1), it is reasonable to hypothesize that distinct mechanisms of carcinogenesis and/or progenitor cell types are more susceptible to the impact of sex. In SHH medulloblastoma, the cell of origin is hypothesized to be the cerebellar granule neuron precursor that may be particularly susceptible to *TP53* mutagenesis (1, 42). In our study, we observed the highest number of sex-DMPs in SHH in which females had worse outcomes than males. Whether these sex differences in epigenetics of SHH are a coincidental product of the cell of origin or themselves drive outcomes remains to be investigated in other studies. During early development, sex hormones enact vast changes in epigenetics that determine sexual phenotypes (11). Using *in vitro* and *in vivo* models of medulloblastoma where sex of the host and tumor is known may help to further uncover sexually dimorphic biologic mechanisms of medulloblastoma development.

Though we have a large sample size and a validation cohort with which we conducted sex-stratified analyses within medulloblastoma subgroups, our study is not without limitations. While medulloblastoma is the most common malignant brain tumor in children less than 19 years of age (1), the parent study was not conducted exclusively in children and limiting it to children would have greatly diminished our sample size. It may be that pediatric and young adult medulloblastomas have different sex-specific methylation profiles and this should be investigated in appropriate studies in the future as there are endogenous changes that occur between childhood and adulthood that may impact tumor etiology. While the parent study had various clinical data, including survival, we are lacking risk factor data such as birth characteristics and other exposures such as radiation (43), which are hypothesized to impact medulloblastoma risk. We cannot rule out the possibility that the lower survival reported here in females versus males in SHH tumors may be due to subsequent cancers or long-term adverse events rather than tumor progression, as this data is also lacking. This study did contain gene expression data from microarrays, but there were few sex differences in gene expression (results not shown) identified in our initial analyses. RNA sequencing data with greater breadth and depth of gene coverage may allow for the identification of gene expression differences that could further help identify biologic mechanisms underlying sex differences in methylation and medulloblastoma tumorigenesis as we have reported previously in osteosarcoma (44). Additionally, single cell RNA sequencing and tumor microdissection might further highlight sex differences in medulloblastoma genomics as has been observed in adult glioblastomas where sex differences were found to be dependent on the sex chromosome composition of the tumor rather than the host (45). Sex differences in treatment received or response to therapy (46) may underlie the observed sex differences, particularly in SHH medulloblastoma, but this information was not available for evaluation herein.

To conclude, in our sex-stratified analysis of methylation differences within medulloblastoma subgroups, we identified sex-DMPs that varied by subgroup with SHH having the highest number of DMPs. Interestingly, in this study we only observed sex differences in survival in SHH medulloblastoma where females had worse long-term survival than males. We found 10 genes with DMPs that were conserved across subgroups suggesting a shared genetic background by subgroup may underlie some of the observed sexual dimorphism in medulloblastoma. Pathways identified within subgroups were largely signaling pathways including TGF-β, neurotrophic receptors, and NOTCH, which are known to impact prognosis in medulloblastoma and according to our findings may vary by sex within subgroups (34, 47, 48). Importantly, we identified four genes that housed sex-DMPs that also have chemotherapies available that could be studied in a sex-specific manner to improve outcomes for males and females with medulloblastoma.

## Data availability statement

Publicly available datasets were analyzed in this study. This data can be found here: GEO Series GSE85218 - https://www.ncbi.nlm.nih.gov/geo/query/acc.cgi?acc=GSE85218 GEO Series GSE93646 - https://www.ncbi.nlm.nih.gov/geo/query/acc.cgi?acc=GSE93646 GEO Series GSE134379 - https://www.ncbi.nlm.nih.gov/geo/query/acc.cgi?acc=GSE134379.

## Ethics statement

As this is publicly available data the study does not require consent for participation.

## Author contributions

NS, RM: Data analysis, results interpretation, manuscript drafting and editing. LM: conceptualization of study, study design, data analysis, manuscript editing. LS, DL, CM, TH: conceptualization of study, manuscript drafting and editing. LW: study oversight, conceptualization of study, study design, data analysis, manuscript drafting and editing. All authors contributed to the article and approved the submitted version.

## Supplementary material

Supplementary Figure 1 Heatmap showing methylation levels (row-scaled β-values) of statistically significantly differentially methylated positions (DMPs) by sex (adjusted p<0.05) from the autosomes in Newcastle cohort within subgroup **(A)** SHH, **(B)** WNT, **(C)** Group 3 and **(D)** Group 4. **(E)** The number of genes that contained a DMP by sex for each subgroup and the overlap of those gene sets.

Supplementary Figure 2 The number of sex-DMPs for each SHH subtype and the overlap of those probes.
