# Supplementary figures and images for: Sex differences in methylation profiles are apparent in medulloblastoma, particularly among SHH tumors
Source: Front Oncol. 2023 Mar 24;13:1113121. doi: 10.3389/fonc.2023.1113121 (PMC10080161; doi:10.3389/fonc.2023.1113121)

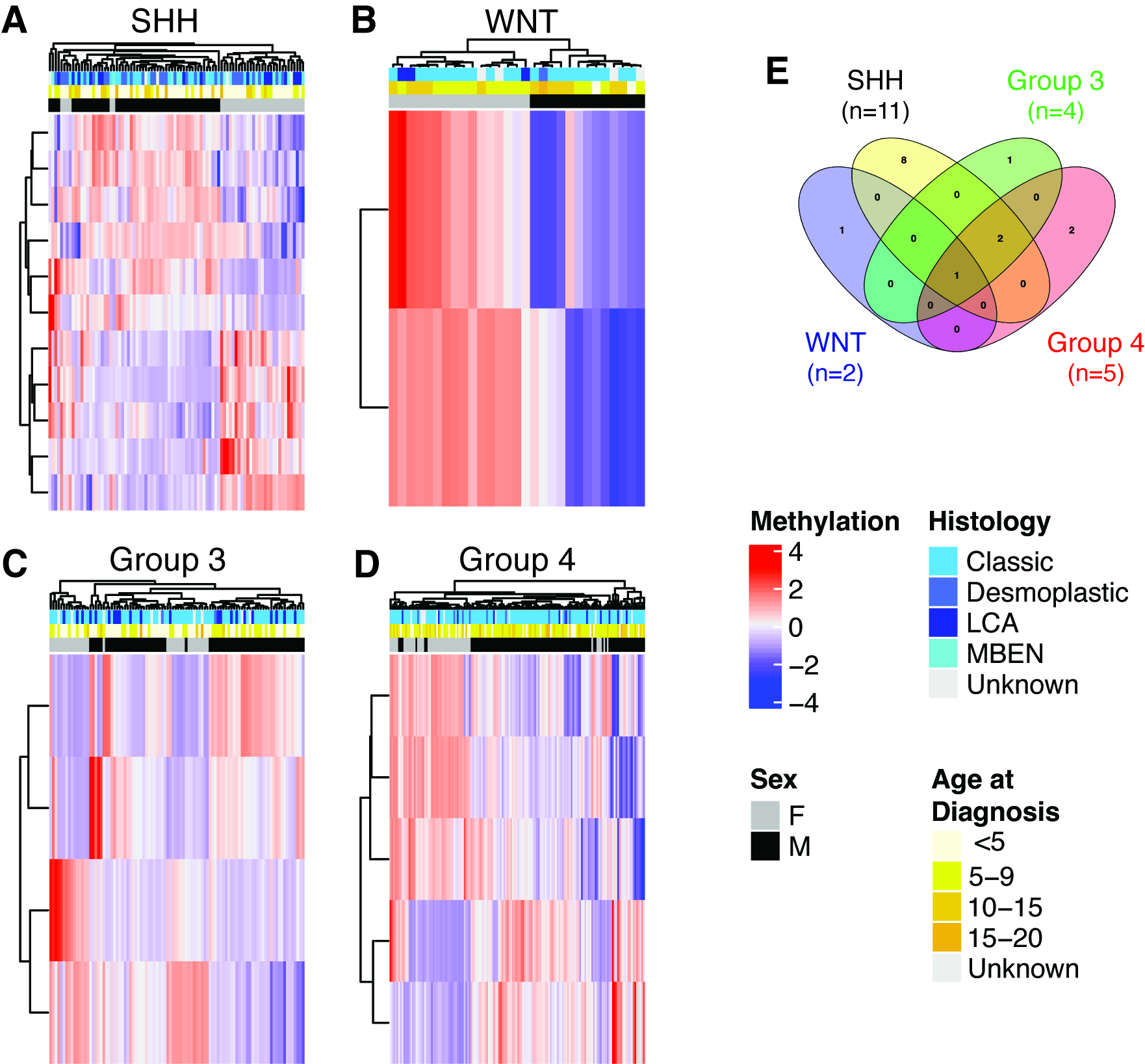

Supplement: Supplementary Figure 1 — Heatmap showing methylation levels (row-scaled β-values) of statistically significantly differentially methylated positions (DMPs) by sex (adjusted p<0.05) from the autosomes in Newcastle cohort within subgroup (A) SHH, (B) WNT, (C) Group 3 and (D) Group 4. (E) The number of genes that contained a DMP by sex for each subgroup and the overlap of those gene sets. [file Image_1.tif]

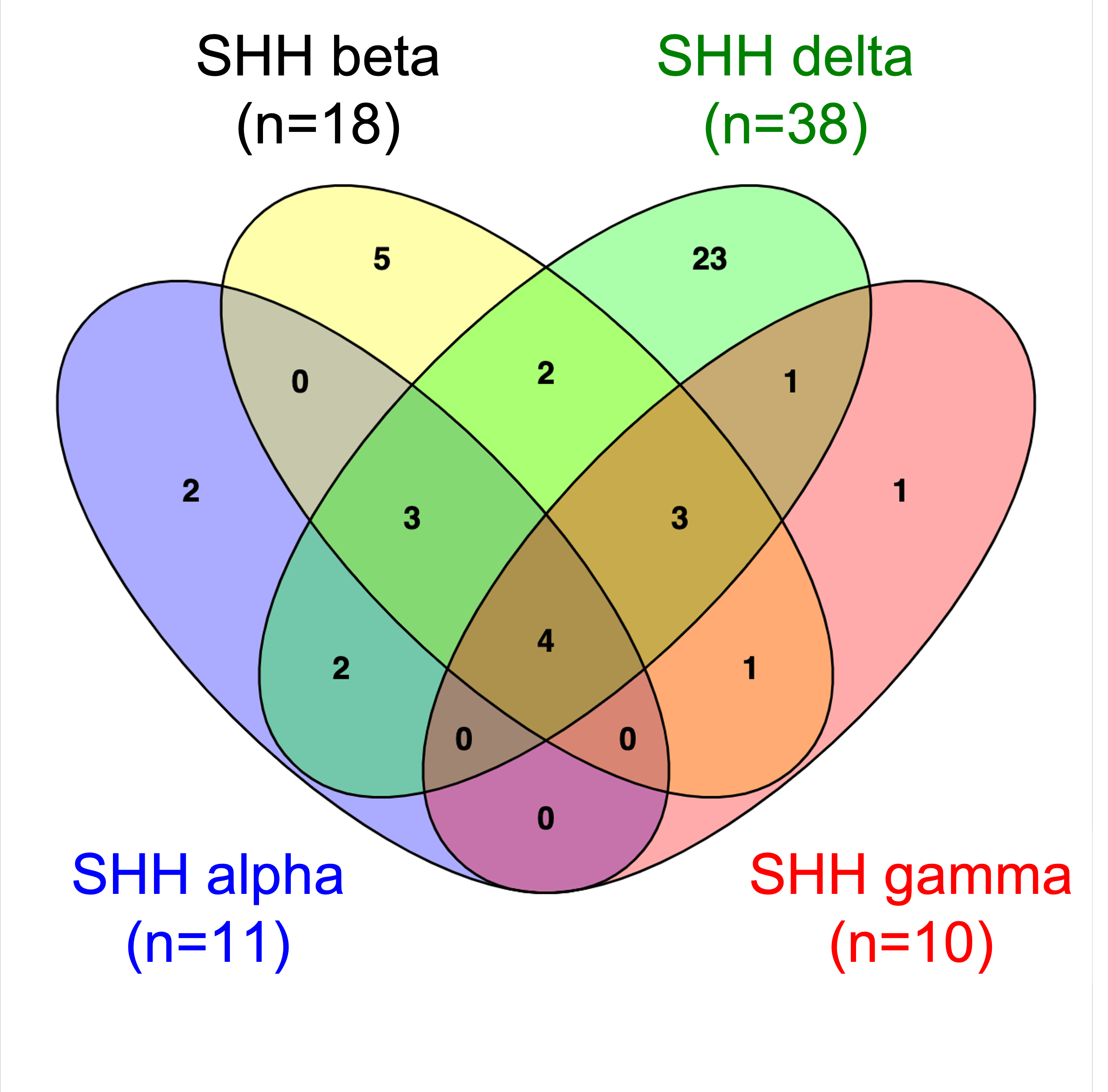

Supplement: Supplementary Figure 2 — The number of sex-DMPs for each SHH subtype and the overlap of those probes. [file Image_2.tiff]
